# Odontological Society of Pennsylvania

**Published:** 1894-09

**Authors:** 


### ODONTOLOGICAL SOCIETY OF PENNSYLVANIA.

At a regular meeting of this Society, held March 10, 1894, at 1228 Walnut Street, Philadelphia, President Darby in the chair, Dr. C. N. Peirce read a paper on "Pyorrhœa Alveolaris," and at the close cited two or three cases confirming the statements contained in the paper.

(For Dr. Peirce's paper, see page 501.)

### DISCUSSION.

Dr. Brubaker, on being called upon by the President, said, "There is nothing, Mr. President, that I can add to the suggestions made by Dr. Peirce either in reference to individual or to dietetic treatment; both are based on the well-recognized lines of treatment long since found to be efficacious in the forms of the gouty diathesis. The question has been asked why only the meats are excluded from the anti-gout diet and not vegetables also, especially as some of the latter contain proteid matter in large amounts. The only answer that presents itself, is that the albuminous molecule of the vegetable foods is less complex and not so difficult of assimilation."

In answer to the question of Dr. Kirk as to what was the peculiarity of beans that they were excluded, Dr. Brubaker stated that it was largely on account of the percentage of proteid matter which they contain, and which amounts to 23.7 per cent., though it must be remembered that the proteid matter is not true albumen, but a globulin, and, therefore, not as objectionable as a more complex form of albumen.

*Dr. Bonwill.*—Before I speak upon this subject I wish to make some remarks upon a charge made at a recent meeting by one of the speakers, a guest of the Society, which, owing to my deafness, I did not hear. My views in the paper read were regarded as "elementary." I laid no claim to the matter therein contained as being scientific, but it comprised the substance of what I knew and practised. His remarks on that occasion were unauthorized and unbecoming.

I am not on record for having said one word upon the subject of this evening's essay, notwithstanding it has figured for at least twenty years as not only an incurable disease, but its cause has never been satisfactorily explained. My silence is not that I have had no subjects for treatment, nor that I have no views upon it worthy of notice.

I presume I have had about as varied a practice as any one here, and, while my opinion may be regarded as of little value, yet I certainly have some right, after an experience of nearly forty years, to an opinion. What I claim to know can be demonstrated, and the result of that experience can be shown in the living subjects. Many of us may not be so profound in chemistry—organic or inorganic—as our essayist, yet with the assistance of others learned in the law of chemistry, we may reach nearer to a satisfactory explanation. I am no chemist, and few, if any, of our clan practically know any more.

It is possible that some persons who are troubled with pyorrhœa (I retain the old nomenclature as good enough for me) have gouty diathesis. It may be I do not know the symptoms of gout. There is one thing I do know, however, that when I can take the many cases that have come to me from the practice of the so-called best men in our profession, who have given them up and declared that no one could save such teeth, and restore those teeth to usefulness without constitutional treatment, and have only seen them twice or thrice at most, the gout has not been in my way. If it be a disease, then every part of the membrane should show it and the deposit take place from the very apex of the root; or, more properly, all portions of the tooth's membranous structure simultaneously should be involved. I have yet to see the first tooth where a deposit has been found at the apex or on any portion of the root without the loss first of the membrane at the cervix. Any one who will with exploring needle search each case will see that when a secretion is pouring forth from about the cervix, accumulations of calculus will be found only on the parts where there is no healthy attachment of the investing membranes.

I can understand how the gouty diathesis can be a factor by depositing surplus calcareous salts found in the blood in the mouth, through the numerous glands emptying thereinto, and mixing with the large amount of starchy matter from the food and the mucous secretion from the gums, as the formation of calculi in the gall-ducts and bladder, but never as a predisposing or exciting cause of pyorrhœa. That there is a surplus of uric acid in the blood of gouty patients admits of no conjecture, as I have detected its peculiar odor in the breath and when drilling into the pulp-canal to relieve pus accumulations. If it should occur at the apex first, then all pulps of such teeth would soon become involved, which is not true, however.

Hundreds of cases have I seen where one side of a root has been involved and stripped of its investments to the very apex, and the pulp at that extremity finally become affected and death ensue.

We often see one of three roots of an upper molar with accumulations, and have cut that entirely free from the others, and they never became affected. It is my opinion that no such accretion could be formed by either investing membrane.

Then you would ask how I treat all my cases; if there is any special way that gives me success where others have failed? First let me say, that in my original practice, or where I have had charge of any mouth from childhood, I do not know of any case where pyorrhœa has ever had a foothold and the teeth lost from such cause. I am aware that this is a bold assertion. But, when I can say that many who commenced with me years ago still make their appearance, some yearly, others not so often, I have no cause to regret what I have done.

Before I speak of the treatment, let me call attention to one factor that has never been noticed, so far as I have kept up with the literature of the subject. False articulation has much to do with the cause. The extraction of one or more teeth early in life makes such a change in the relation of the whole denture that the lateral and direct force brought to bear upon one and all, especially the molars and bicuspids, causes the investing membrane to be unduly forced or strained and its function materially lost. The extraction of the first permanent molar in the lower jaw causes absorption of a large portion of the alveolar process on the roots of the adjoining teeth and the gum still in place, yet loose about the cervix, and for half an inch under, where accumulations are found long before any other place, and from this the teeth adjacent are involved. But this is little, indeed, when the whole denture is thrown out of gear, as cog-wheels become by wear and change of relationship. The best evidence I can give of this fact is, that before I resort to any treatment, I invariably look at the articulation and take an impression of both jaws and place them in my articulator, which reveals just what I see in the mouth,—the teeth most at fault.

So long as this is allowed to remain, no cure can ever be affected. Take off the calculus, and still this cause remains to condemn you for your want of vision. As soon as one tooth becomes involved more than another, the offender is at once thrown from the socket and articulation is thrown largely on this one and its antagonists, and the disease becomes magnified until the tooth is lost. Could you have seen this, and with your engine and corundum wheel have placed this tooth and its opponent in harmony, as you would shove a cog out of place, or one that is loose tighten and make shorter, every time there would have been perfect success. I have often seen cases given up as hopeless by others, when by simply rearticulating them, they have been restored to perfect health for years and with no loss or extension of the disease to other healthy teeth.

So often in mouths where the teeth are far beyond the average, and scarcely a decay to be found in any of them, have I had to combat with loose teeth ; not the whole set, but an individual tooth and its antagonists. On questioning the patient I have found that biting off threads or the finger-nails produce the effect of sharper articulation. I simply shorten these teeth, so that they cannot longer use them, and all is well.

A few weeks ago I had a patient whose teeth were given up. They were almost faultless and placed in the process firmly as rocks, save three or four. She told me on inquiry that it was her habit to crush ice between those teeth, and she could not think she was injuring them, and her dentist never asked her as to what she was doing with her teeth. One, or perhaps two, I made her lose. She had very little tartar upon any of them, not even upon those extracted.

Take another case : A man who had lost nearly all his bicuspids and molars, except where it was hit and miss, and of no value for articulation, came to me with the left lateral incisor, lower jaw, so tender that cold no longer affected it, but hot water gave intense pain. It had been this way several days. My first thought was the pulp is involved; but there was no decay. Then I looked after the articulation, and I found that nearly or quite all the incising and even grinding was done upon this one tooth and the two upper antagonists, that were much larger and fared better. The first treatment suggested to me was to drill into the pulp-canal and remove the congested membrane. But I simply cut from the labial surface of the inferior lateral and the lingual surfaces of the superior antagonists, and the pressure was instantly relieved; and without even a counter-irritant the tooth began at once to improve, and from that time was no longer a cause of irritation. There was no accumulation on it.

To further illustrate how far false articulation is a factor in the loss of good teeth, some with loads of tartar on them and others none, I will recall a case of a desperate drunkard who had been my patient for years, and there never were any signs of loosening of the teeth and no previous signs of tartar or calculi. In one of his fits he caught foul an inferior lateral incisor, right side, and in the spasm of his jaw fractured the process on the lingual side. When he came to me for treatment, three weeks after, there were already accumulations taking place on the root, and the crown was quite out of its place and was struck at every motion of the lower jaw. At this time pus was pouring out from every surface, and no shortening of the crown and ligating would retain it and cause it to grow firm again. I had to remove it. There can come a shock to every tissue, bony or soft, that means death whether the system is in health or not. But the bulk of such cases can be saved by regarding articulation and very little interference on the part of the surgeon.

I could multiply these cases and their cures. One more, however, I must notice. One of the upper classes came to me eight years ago in great trepidation. He informed me he had been to not only four or more of the so-called best dentists of Philadelphia, but to prominent physicians and surgeons, and each and all decided there was no treatment which would reclaim those remaining, but one by one they would leave him.

His teeth were as nearly perfect as anything I ever saw,—only one filling of oxyphosphate, and it was to cap a pulp, which was one of those he lost. Two molars in the lower left side and one in the upper, same side, I refused to treat at all. The rest I cleansed, which was done at one sitting, having him two successive sittings to see if any small particles remained and to complete the articulation of those thrown out of position; and that mouth stands today as I left it. He comes to me every year, and it requires but one sitting. This gentleman from high living should have been gouty. But, if he was, it did not interfere with the treatment.

Allow me to speak now of another factor which dentists will be reluctant to admit. You may not have thought that bad dentistry has anything to do with this trouble. Well, I am sure it has. If gout has so much to do with this frightful disease, how is it I can take a mouth filled with flat approximal fillings leaving at least twenty spaces between the teeth, and then by keeping them all apart with pink gutta-percha until a proper width is attained, the whole jaw becomes at once easy and quiet, and the teeth again assume their normal position in articulation with their antagonists.

Finally, as the spaces are wide enough to permit the alveolus and gum to get and retain their normal status, I make contour fillings that buckle up to each other so that no food can possibly pass. Try what I say, and you can do what others have not done. I see contour work, but when it is investigated the teeth were once cut away and allowed to come together; and, instead of taking time to force them apart with the gutta-percha and let the teeth assume of themselves normal articulation, a separator is placed in and they are filled at once, and the gum and process are left pinched and will never be as healthy and firm as when wide apart; besides, the crown fails to assume the normal articulation. Again, when these ground arches are broken into by extraction, the teeth are never so firmly in contact as to prevent food from being driven between them and causing irritation and accumulations, as well as irritation from undue pressure. The teeth will stand an immense deal of abuse, as bad dentistry will testify; but modern life with its soft food and sweets, with no true legitimate work given to the teeth, weakens the tissues and causes absorption of alveolus and makes the teeth loose, and the gums will not close round them any longer, and secretion will accumulate and pyorrhœa step in, but not true disease. Lastly, artificial teeth are no mean factors in causing destruction to the remaining teeth. Look at the bad-fitting plates with their borders allowed to come in direct contact with the cervix of every tooth, for fear it will not be retained; sharp edges to irritate and loosen the teeth in contact. And with bad articulation they are more an apology for a substitute than a realistic set. They do not relieve the work to be done on the few remaining teeth, but force them out of position and cause weakening of the tissues surrounding.

Look at the mischief that crowning does with a band as an irritant in so many cases, and this extending to others causing secretion to accrue, and finally loss. When properly done and the teeth wedged tightly in, they are a salvation; and how many cases of loose roots can be restored to firmness if well done by crowning, by giving them work to do. See how much mischief is done to the gum-tissue that spreads far and wide from the use of arsenic on the pulps. I will not multiply cases that I know to be factors in this dreaded disease. Have well-adapted instruments and keep them sharp. Use a dull bur in the engine. Do not be afraid of hurting the patient, fearing they may not return. Take the bulk of secretion off at the first sitting, and be sure of it. Cut all loose and excess of gum down to healthy tissue, or rather sound borders, leaving no cause for deposits. It is worse than useless to allow it to bag around the cervix. Make the gums irritable, and they will grow stronger and grasp their fellows with firmness. Use carbolic acid, creosote, or anything that will act as an escharotic, and burn the dead tissue of the membranes. Have the patient come back in one week, and you can tell if any secretion remains. Remove at once and not disturb the healing tissues. I told you first of all, restore the articulation. Let the parts heal in their own blood, and do not interfere every day with nature.

In other words, imagine you have a fistula to treat. You know the walls will not come together and heal unless they are excoriated and freshened with the scalpel or an escharotic; and so here don't be afraid of tearing the membranes off the tooth or the investment. If I can put the actual cautery there, so much the better. Look on the space between tooth and alveolar border as a narrow fistula. And when clean and well burnt out, it will be sure to heal. If the surface of root at any point is rough, bur it off, and also the dead or lifeless alveolus. I have never cut too much, but frequently too little. The treatment to be given them after leaving you is simple. No washes even. Simple tooth-soap, with the finest pumice in it, is all I use. As small a tooth-brush as is to be had and with not more than three rows of bristles lengthwise, and the whole brush not over one inch long, or an inch and a quarter. One minute brushing immediately after eating. Not more. Never use the brush across, nor up and down, but put it in one place and press down upon the teeth and wriggle it around, keeping it pressed between the teeth.

A small brush requires less pressure to work in between the teeth when the bristles are far apart. Have the patient see you at least every six months, and then do not unnecessarily cut the gums loose, but gently run a scaler around and let them go.

Last, and not least, a tooth-pick. What kind? Never a wooden one of any shape or fibre. They drive the gum up and away from the cervix and keep it forever loose for secretions to embed there. A quill is better than all else, except a very narrow, thin, and long gold one.

In the use of the silk floss charge them not to "see-saw" with it and keep the gums always cut away from the cervix. It does immense mischief to the gums. Pardon me for this lengthy discussion. As I have never said anything before upon it, I say now and for all time, I am satisfied as to the simple treatment of common sense and have no cause to regret the past, and see none why I should change it for all the theories and conjectures of professors or chemists.

*Dr. Broomell.*—I became so interested after listening to the discussion at our last meeting, that I resolved upon making an investigation myself. Since that time I have made it a part of my daily practice to question those patients in whose mouths I noticed the least inclination to pyorrhœa. The result has been that out of ten patients whom I questioned, all but two reported a rheumatic or gouty diathesis. Two of the cases were especially interesting to me, from the fact that they represented the two extremes in the habits of life and in the manner of living. The first one, a lady, about fifty-five years of age, was a "high liver" in every sense of the term. Entertaining and being entertained, breakfasts, dinners, and teas following each other in rapid succession, partaking most generously of everything that would please the palate and lighten the heart.

This much of her history was given to me without my having to ask for the information. Upon examining her mouth, I discovered aggravated pyorrhœa about several of her teeth. When I inquired into her general health, she informed me that she was a terrible sufferer from rheumatism, gout, and heart-disease. I also learned that her father had suffered in the same manner, and had lost all his teeth prior to his death, no doubt from pyorrhœa, by the description given me by my patient. Upon examining the smaller articulations, there appeared to be no indications of chalky deposits. This patient, I should think, would be a powerful witness as to the correctness of Professor Peirce's theory.

The other case, a Mrs. H., aged fifty-eight, represented a typical subject of the other extreme. Being a strict vegetarian, she declared that neither she nor her parents before her had ever partaken of animal food, shunning all meats, fowls, game, fish, butter, eggs, milk, and spirits. She had been taught, and in turn was teaching her children, to do all that could be done to redeem fallen humanity. For her, olive oil took the place of butter and lard. Her hands could not withstand the irritation produced by wearing kid gloves. The shoes upon her feet were for the greater part constructed from vegetable products, while bone buttons upon the clothing could never be tolerated. To further show me the regard she had for her belief, she has forwarded me a number of bills of fare at the vegetarian restaurant at which she dines when abroad. If, as I understand it, uric acid is produced by animal food, and pyorrhœa is produced by uric acid, this case appears to be somewhat mysterious. Pyorrhœa existed to a considerable extent. The finger-joints were abnormally enlarged, and the complexion, which one would think should be clear and spotless, was wonderfully imperfect.

What, then, can be the conclusion when we find pyorrhœa in two subjects so extremely different.

The President next asked Dr. Brubaker to tell the Society about gout and gouty diathesis in the matter of cases of pyorrhœa.

*Dr. Brubaker.*—Mr. President, it would be impossible in the short time at my disposal to discuss adequately the causation or pathology of gout and its relation to pyorrhœa. As I am, however, to a slight extent, responsible for some of the statements in Dr. Peirce's paper, I will take the opportunity of replying to some of the criticisms which have been made to those statements and which may have a bearing on the pathology of gout and pyorrhœa.

Notwithstanding all the theories which have been propounded from time to time by pathologists as to the cause and pathology of gout, there is yet much difference of opinion; but as to one factor most observers are in accord,—viz., that the underlying cause is the presence in the blood and tissues of uric acid. Inasmuch as phenomena of general gout are so extensive and complex, it has been deemed necessary by some pathologists to invoke the aid of other factors. Dr. Duckworth, for instance, has been at great pains to illustrate the influence of the nervous system in the production of some of the curious phenomena. Nevertheless, this eminent authority states that while "the peculiarly presented relations of uric acid, even in true gout, do not constitute the whole of the disorder," yet, "in spite of teachings to the contrary, I would affirm at the outset that this part of the pathogeny of gout is certain, so that it may be plainly stated, 'no uric acid, no gout.' I would express my adherence to the view that the most unequivocal evidence of true gouty disease is that derived from the presence of uratic salts in the tissues." (A treatise on gout, p. 15.) A statement was made this evening that the uric-acid diathesis and the gouty diathesis are not synonymous terms. This distinction may be made, but in view of the statements of Dr. Duckworth just alluded to, it is hardly tenable. It is true that uric acid may exist in the blood without giving rise to any particular gouty manifestations that can be recognized, but it must be admitted that in the vast majority of instances there is a close relation between that type of disordered nutrition known as the uric-acid diathesis and that type of constitution known as the gouty.

It has also been asserted here that gouty attacks can be produced without uric acid being present, at least in excess. This is exceedingly doubtful. This idea originated with Dr. Todd many years ago, but it has never received much confirmation.

One of the speakers this evening, Dr. Allan, quoting from an article in Pepper's "System of Medicine," vol. ii., said that the distinguished writer entertained the idea that uric acid is not the cause of gout. Yet an attentive reading of the article *in extenso* will reveal the fact that the writer regards uric acid as the most constant and important factor in gout production, though he does state that it does not explain all the phenomena. The treatment, both medicinal and dietetic, is based on the view that uric acid is largely the exciting cause. An apparent contradiction to the views of Dr. Peirce that a highly nitrogenized animal diet is a necessary antecedent of the uric-acid diathesis, was offered by Dr. Broomell,—viz., that pyorrhœa is seen in vegetarians and in those who consume but small quantity of meat. This is, indeed, true. But it must be remembered that uric acid may arise from a disintegration of the proteids of the tissues, as well as from an imperfect assimilation of the proteids of the food. And if there is an excess of proteids in the vegetable foods or a defective power of elimination of waste products by the kidneys, it is conceivable that uric acid, one of the products, might be retained in the blood and give rise to the characteristic phenomena.

Again, the chemical analysis of the deposit has been called in question, and it has been asserted that no uric acid, or but little, is present in any given deposit. This is no doubt true in some instances. It must be remembered that in aggravated cases of pyorrhœa there is a continual discharge of pus, and the uric-acid salts are carried away with it. It is not to be expected, therefore, upon the removal of a tooth to find large masses of pure uric acid. The deposit from any tooth is largely composed of an albuminous basis, which, upon the addition of nitric acid, becomes of a bright yellow color. This often obscures to a marked extent the color of the murexide test. The albumen also interferes with the crystalization of the uric acid. Again, care and patience must be exercised in examining the deposit for uric acid. In an analysis made to-day, two hours were occupied with one specimen, from a few grains of which eight crystals of uric acid were obtained. This may not be very striking, but it is sufficient to indicate its character.

Another objection that was made was that pyorrhœa patients are not gouty patients. The value of this statement will depend upon the knowledge and experience which we as individual practitioners have had of determining that fact. How many practitioners here would have been able to diagnose the gouty character of the disordered heart in the first case alluded to by Dr. Peirce? Yet this was done by a most distinguished clinician in this city, and had the patient followed the course of treatment suggested, it is quite likely he would never have had pyorrhœa.

The view first enunciated by Dr. Peirce as to the pathology of pyorrhœa, as published in the INTERNATIONAL DENTAL JOURNAL for January, 1894, may or may not be true; but there is a strong supposition that it is, and the truth of that supposition can be determined by the result of treatment. If these pyorrhœa patients get well on an anti-gout treatment, it is presumptive evidence that they were gouty and the pericementitis but a local manifestation.

*Dr. G. S. Allan.*—I have listened to this paper with exceeding interest. I hoped to get some new points; I haven't got any, except as to treatment, which was general and indicated in Dr. Peirce's former paper. I confess I feel a great deal as if I had been in fairy-land, and that I had listened to some particulars of a disease of which, so far, in my own practice, I had known little or nothing about. The statements in that paper, if uncontradicted, would go to prove that the profession heretofore had practically no knowledge of what pyorrhœa alveolaris was and no knowledge as to its etiology, and that the treatment heretofore on the lines that had been adopted ought to have been, if they have not been, futile and unavailing.

I questioned the accuracy of Dr. Peirce's statements and conclusions. First, on the ground that the presence of uric acid in the system, or uric salts, did not indicate gouty diathesis. I quoted from Pepper's "System of Medicine" an article by Dr. Draper on Gout, strongly taking up the position that I had held. I also read a quotation from the *American Journal of Medical Sciences* from a later writer, taking identically the same position. To-day I called upon my old friend, Dr. Thomas Morton, of Philadelphia, and I asked him the same question. He corroborated my position and statements, and when I asked him if I could quote him, he said, "Certainly;" which I now take the liberty of doing.

Taking it for granted there is anything in this theory,—which I doubt,—there are no proofs that the uric-acid and the gouty diathesis can be considered as one and the same, but Dr. Peirce used the two terms almost synonymously. I stated before that you could have gout without uric acid in the system, and you could have uric acid in the system and no gout; and I believe my statements can be well borne out by the evidence of able practitioners.

In a paper I read before the combined societies of New Jersey and Pennsylvania at Asbury Park a few years ago, I made this statement, that if a practitioner had charge of a patient's teeth, and the patient was conscientious and carried out directions fully and as indicated, it was inexcusable that pyorrhœa alveolaris should have its origin in that mouth, and certainly inexcusable that any destructive work should be done by the disease. That statement I still adhere to; and in that statement I absolutely ignored any such thing as constitutional treatment. In the analyses that I had made of this hæmatogenic calcic deposit, absolutely nothing but a trace, and a trace only, of uric acid was found in that deposit. Now, that is significant. In these analyses that Dr. Peirce has made, the doctor draws attention to the uric acid found in minute quantities only, ignoring the fact that the great bulk of these deposits are calcium phosphate and calcium carbonate. In none of the analyses heretofore made has uric acid ever been recognized, nor was the presence of uric acid in these deposits ever suspected till Dr. Peirce made his report. Now he comes forward and his analyses seem to prove that uric acid is a predominant element. I don't believe that a quantitative analysis made of this hæmatogenic calcic deposit would have shown anything more than a mere trace of uric acid, and this I say because I have the results of the analysis Professor Ricketts made, and from such a large quantity of the material, and failed to find anything but a mere trace.

Another point I made, and that was that after the deposit had been formed no constitutional treatment could take up that deposit, carry it back into the blood-current, and have it eliminated through the ordinary excretory channels. Now, there is no such thing,—there can be no such thing,—from the very nature of the case. Here is a deposit, hard and insoluble, deposited against the root of the tooth. The blood cannot get at it in any way whatever, and I cannot conceive the possibility of any constitutional treatment removing that deposit. The mere statement of the conditions makes the impossibility self-evident. But supposing a good portion of that deposit, as Dr. Peirce indicates, is composed of urates, those urates are still simply mechanical irritants, situated as they are. They are in a place where they act mechanically, and mechanically only; and I maintain that all this train of hæmatogenic calcic pericementitis symptoms comes from the irritating effects of the deposit acting locally."

*Dr. Kirk.*—How came the deposit there?

*Dr. Allan.*—I never denied that a constitutional condition was at the bottom of the deposit. But that is one thing; the disease itself is another. As I stated, the stone in the bladder or in the urinary ducts comes from some constitutional condition; but when it is in that position, then it starts in business for itself, and all the inflammatory conditions that follow are due to the deposit and not to the constitutional diathesis or condition of the system that produced the deposit. With every desire to do so, I fail to see what point or what bearing this theory will have upon the treatment of pyorrhœa alveolaris, and for these reasons; summing it up, it is just this: Uric acid is not a necessary concomitant of gout or rheumatism; and, granting even that it is, the train of symptoms we have are local manifestations of a local irritant. I do not deny—I never have denied—that there are cases of pyorrhœa alveolaris that, so far as we know, are not caused or produced by a deposit of any kind. I have had such cases in my own practice more than once. I could not account for them, but that does not prove that the great majority of the cases are not produced by this hæmatogenic calcic deposit.

Since I was here last month I made it a habit of asking almost every patient whether they had pyorrhœa or not; have you gout or rheumatism? Tabulated and put into shape for drawing deductions, I find that the results are worse than conclusions. In other words, gouty patients had no pyorrhœa, and patients that had no gout or rheumatism had pyorrhœa, and the significance of the gouty diathesis was simply visionary.

There is a difference between a deposit made in a tissue and one outside of a tissue. The deposit that is on the tooth is outside of the tissue, not inside. It is not, so far as we know, in the peridental membrane. It is a deposit on the exposed surface of the root of the tooth, being deposited on the surface of the root only. Outside of any possible influence the blood may have, I don't see how constitutional treatment can absorb or in any way dispose of that uric-acid deposit.

Where there is death of the periosteum and we have a deposit on the denuded surface of the bone, I do not see how any constitutional treatment would be available for removing the deposit. If Professor Peirce's theory be correct, he has not made it plain, if this deposit takes place in the pericemental membrane, how it is transferred from the pericemental membrane, dead or living, on to the surface of the root of the tooth where we find it.

Dr. McQuillan inquired of Dr. Allan if, in speaking of treating pyorrhœa, he meant that a cure was effected, or whether it is simply the arrest of the disease. Dr. McQuillan acknowledged that he could not cure it.

*Dr. Allan.*—I will answer Dr. McQuillan by referring him to my paper, in which I said that I could not do it, except in some cases. If the disease is taken in its earlier stages a cure can be effected, but when it has passed beyond what we call the earlier stages, it seems all we can do is to alleviate the consequences; but to make a radical cure in a pronounced case where any quantity of the peridentium has been destroyed, I think most difficult. We can save the tooth for a long time, but that does not cure it. That is arresting it.

*Dr. Truman.*—I desire simply to say that I have cured exactly such cases; cured them perfectly and kept them there.

*Dr. Kirk.*—Allow me to make an explanation. Since the reading of Dr. Peirce's paper at the last meeting, I have been employing all my spare time in making some chemical investigations of these deposits, and the first tests I made were all from cases carefully selected with reference to their possible gouty association.

There is one factor which will necessarily modify the results which one may get from a test of these deposits, and that is the question of quantity. The quantity of material at our disposal in any single case is, necessarily, extremely small. I have examined some seven or eight specimens secured from mouths which had a definite gouty history. The first three of these I put through a series of careful tests and got some slight reaction, but one which I would not like to take into court as definitely showing uric acid. The other cases were those in which I had larger deposits, and were treated not only by the usual test, but they were carefully tested from every stand-point possible, in order to determine whether they did or did not contain uric acid, and I found that they did contain uric acid. In these several specimens there was a marked difference in the character of the deposits.

Some of them were extremely adherent, and were such as Dr. Allan has alluded to as being in close contact with the cementum. I believe they were in close physical union with the cementum of the tooth, with no pericemental membrane intervening. I had two cases, however, in which the deposit was, as nearly as I could determine by the unaided eye, located in the pericemental membrane, because the deposit easily peeled off with a small strip of the membrane adhering to it, showing it was situated in the enveloping membrane of the root. These two cases gave the most marked reaction for uric acid. It was not a haphazard difference, but a very marked one both as to quantity and intensity. It seems to me possible, in fact, highly probable, that deposits in close organic connection with the cementum, and in which the pericemental membrane has been destroyed, are those which mark a later stage of the disorder. The two cases of which I speak, in which the uric deposit existed only in the pericemental membrane, had not gone on so far, because the deposit of serumal tartar was relatively slight in quantity, with urates in relatively greater proportion.

I want to state also that all the deposits I examined, with the exception of the two in the membrane, consisted essentially of phosphate of lime, and that the uric acid was small in amount. In this relation let me call attention to the analogy which these deposits present to vesical and renal calculi. Cranstoun Charles quotes Thudicum as stating that in nearly all of the vesical and renal calculi examined, of those which contained uric acid, it existed as a nucleus around which the calcium phosphate had been deposited in connection with the organic matrix, and existed in layers concentrically arranged. I believe it is well recognized that all vesical and other calculi must have, or are likely to have, some nucleus around which the deposit takes place, and in many this nucleus was found to have been uric acid. The chemical and physical conditions are such that we might reasonably expect the same chemical and structural features in the deposits of serumal tartar.

Dr. Guilford inquired how the deposition found in the membrane could afterwards be transferred to the cementum.

*Dr. Kirk.*—I do not know that it would, except by production of inflammatory action. A denuded area of cementum would itself be a source of irritation, and the irritation would invite further deposits. I should look upon the deposit in the membrane as precedent to the deposit on the root.

It seems to me that some confusion has arisen in this discussion from the failure to clearly distinguish between the exciting cause of pyorrhœa, which is undoubtedly the deposit, and the predisposing cause which gives rise to the deposit, which Dr. Peirce believes to be the uric-acid diathesis. This seems to me to be correct and logical reasoning, in view of the facts respecting the character of the deposit and the results of specific systemic treatment. Local treatment alone will not cure the disorder; if we hope to prevent recurrence, we must remove the predisposing cause.

*Dr. Bonwill.*—I wish to correct a statement I made in my paper, to the effect that constitutional treatment should never be used. At least one in every two persons has some derangement of the gastric function, which leads to other troubles. Instead of giving remedies, keep the stomach all right by proper food; never drink when eating. That is all the treatment I would give. That is constitutional. Give the food in a proper way.

I would propose as the best way of reaching this thing, that we have clinics, and that Professor Peirce shall bring his subjects and let us see whether or not we understand what pyorrhœa is. I don't believe I do. I want to find out, and that is the way to get at it.

*Dr. Peirce.*—My friend, Dr. Allan, placed words in my mouth on two occasions, words that I do not recollect having uttered in my paper or verbally, and that is that I maintain that I induce the absorption of the deposit or the removal of the deposit by constitutional treatment. In every instance where I have spoken of the treatment of pyorrhœa, I have said emphatically that an instrument for removing the deposit was essential. In every case I make that the starting-point, and follow that up by the application of acid carefully applied; and wherever I have spoken of it, I have made that emphatically the beginning of my treatment, and have only contended that in constitutional treatment I facilitate the process of healing. I have not been successful in curing beyond recurrence, but I have been more successful in the last four months, since I used constitutional treatment, than I ever have been before in cases that I have had in hand four or five years and seeing the patients during that time almost monthly.

Dr. Allan replied that he had probably misunderstood the paper.

Adjourned.

